# Effectiveness of a transdiagnostic individually tailored Internet-based and mobile-supported intervention for the indicated prevention of depression and anxiety (ICare Prevent) in Dutch college students: study protocol for a randomised controlled trial

**DOI:** 10.1186/s13063-018-2477-y

**Published:** 2018-02-20

**Authors:** Felix Bolinski, Annet Kleiboer, Eirini Karyotaki, Judith E. Bosmans, Anna-Carlotta Zarski, Kiona K. Weisel, David D. Ebert, Corinna Jacobi, Pim Cuijpers, Heleen Riper

**Affiliations:** 1 Department of Clinical, Neuro-, and Developmental Psychology, Vrije Universiteit Amsterdam, Van der Boechorststraat 1, BT 1081 Amsterdam, The Netherlands; 2 Amsterdam Public Health research institute, Faculty of Behavioral and Movement Sciences, Vrije Universiteit Amsterdam, Amsterdam, The Netherlands; 3 Department of Health Sciences, Faculty of Earth & Life Sciences, Vrije Universiteit Amsterdam, Amsterdam, The Netherlands; 4 Department of Clinical Psychology and Psychotherapy, Friedrich-Alexander-Universität Erlangen-Nürnberg, Erlangen, Germany; 5 Department of Clinical Psychology & Psychotherapy, Technische Universität Dresden, Dresden, Germany

**Keywords:** Depression, Anxiety, Prevention, Internet-based intervention, Cognitive behavioral therapy, Students, Randomized controlled trial

## Abstract

**Background:**

Depression and anxiety are common and co-morbid disorders that affect a significant proportion of students. Innovative prevention strategies targeting both conditions are needed to reduce their health burden and costs. ICare Prevent is such an innovative strategy and contains a transdiagnostic individually tailored Internet-based and mobile-supported intervention. It addresses common risk factors of depression and anxiety as part of a large EU-funded multi-country project* (ICare). Little is known about the clinical and cost-effectiveness of this type of intervention compared to care as usual (CAU) for college students. We hypothesize that ICare Prevent will be more (cost-)effective than CAU in the reduction of symptoms of depression and anxiety.

**Methods:**

A three-arm, parallel, randomized controlled superiority trial will be conducted comparing a guided and an unguided version of ICare Prevent with a control group receiving CAU. The trial will be open-label but outcome assessors will be blinded. A total of 252 college students (age ≥ 16 years) with subclinical symptoms of depression defined as a score ≥ 16 on the Center for Epidemiological Studies Depression Scale (CES-D), and/or anxiety, defined as a score ≥ 5 on the Generalized Anxiety Disorder scale (GAD-7), will be included. Those meeting diagnostic criteria for a depressive or anxiety disorder will be excluded. The primary outcome is change in disorder specific symptom severity from baseline to post-intervention. Secondary endpoints include self-reported depression and anxiety symptoms as well as time to onset of a mood or anxiety disorder until 12-month follow-up. Societal costs and quality of life will be assessed to estimate the intervention’s cost-effectiveness compared to CAU.

**Discussion:**

Transdiagnostic individually tailored Internet-based prevention could be a (cost-)effective approach to tackle the disease burden of depression and anxiety among college students.

**Trial registration:**

Dutch trial register, NTR 6562. Registered on 6 July 2017.

**Electronic supplementary material:**

The online version of this article (10.1186/s13063-018-2477-y) contains supplementary material, which is available to authorized users.

## Background

Depression and anxiety are highly prevalent and debilitating conditions that are associated with considerable economic costs [1–5]. Both disorders have their peak onset in early adulthood, including college years [6, 7]. College students often experience a variety of stressors (e.g. gaining personal and financial independence), which have the potential to trigger or exacerbate symptoms of mental health conditions [8, 9]. Correspondingly, > 20% of all college students suffer from a mental condition, ranking anxiety and depression on top [10–12].

Depression and anxiety during college years have several negative consequences for students and society. For example, a younger age of onset of depression and anxiety has been linked to a more severe and chronic disease trajectory, as well as a higher risk for developing co-morbid psychiatric disorders in adulthood [8, 13, 14]. Moreover, it is clear that anxiety and depression negatively influence academic performance and increase the possibility of college dropout [15, 16]. On a societal level, college students’ untreated depression and anxiety may have significant implications for human capital, specifically when their future employment and income is considered [17, 18].

Preventing the development of these mental health conditions in college students is thus of great importance. Hence, recommendations have been made to implement university-based early detection and prevention programs [19]. These can be distinguished as universal (targeting a population as a whole), selective (providing an intervention to individuals with a specific risk profile), and indicated (targeted to those who already experience elevated symptoms below clinical thresholds) ([20]; p. 20–21). According to previous research, face-to-face college-based universal prevention programs show small to moderate effects in reducing depression or anxiety severity [21]. Yet, data on selective and indicated university-based prevention approaches are scarce, though their application may be particularly effective because they are targeted at those who need help the most ([22], p. 499). Specifically, indicated prevention has been described as the preferred approach based on its potential for detecting and efficiently helping those who have a high risk of developing depression [23].

Internet-based interventions have the potential to fill the gap between the limited university healthcare facilities and the treatment demand [24–26]. In contrast to face-to-face psychotherapy, this approach depends less on therapist availability, offers low-threshold access, and the possibility to use the intervention at any time and place. Moreover, it provides a high degree of anonymity and thus tackles the issue of stigma associated with mental health problems, which negatively affects college students’ help-seeking behavior [17, 18]. In clinical populations, the effectiveness of such Internet-based interventions for anxiety and depression has already been established [27, 28], especially if they are provided with some form of therapeutic guidance [29]. With regard to prevention of depression or anxiety, the evidence regarding the effectiveness of Internet-based interventions is inconclusive. Recent trials comparing indicated prevention programs to controls have shown mixed effects, varying from no differences between the conditions to moderate effects on reduction of incidence rates in favor of the Internet-based preventive program [24–26, 30, 31].

However, to the best of our knowledge, no randomized controlled trial on the effects of online indicated prevention of depression and anxiety has been conducted in a pure college student population. Moreover, past research has largely focused on disorder-specific interventions for the online treatment and prevention of these conditions. Adding transdiagnostic components may address their co-morbidity better, as well as common underlying factors, such as general negative affect and disturbed information processing [32]. Recent meta-analytic evidence on Internet-based interventions has shown that transdiagnostic approaches have no differential effect for anxiety when compared to disorder specific approaches, but they do perform significantly better in the reduction of depressive symptoms [33].

Finally, the cost-effectiveness of Internet-based indicated prevention programs and the added value of human support remain unclear. Though some studies suggest that Internet-based treatment has the potential to be cost-effective [24–26, 34–36], the evidence for prevention is limited. The economic evaluation of the Internet-based prevention program developed by Buntrock et al. [37] shows that such an indicated prevention of depression through the Internet can have a large likelihood to be cost-effective when compared to enhanced usual care. Moreover, although asynchronous support (e.g. by email) has been shown to have beneficial effects on outcomes and retention [38–40], information on the cost-effectiveness of such an investment (e.g. time and costs of eCoach) as compared to no human support for Internet-based prevention of anxiety and depression among students is lacking. Examining further the cost effectiveness of guided and unguided Internet-based indicated prevention in a student population is essential.

### Trial objectives

We designed a randomized controlled trial to evaluate the (cost-)effectiveness of a transdiagnostic individually tailored online intervention compared with care as usual (CAU) in reducing symptoms and preventing the onset of a full episode of depression and anxiety in college students. We hypothesize that participants in the two active intervention groups will experience larger symptom reduction of depression and anxiety than those in the control group. Moreover, we hypothesize that the lesser use of personnel (i.e. eCoaches) in the unguided condition will be reflected in a more favorable cost-effectiveness ratio for this group compared to the guided condition.

This study is part of a large European Horizon 2020 research project (ICare) and will be conducted with similar study designs, procedures, inclusion and exclusion criteria, and primary outcome measures among the general population in Germany, Switzerland, and Spain. The study protocol of the other ICare Prevent trials will be published elsewhere (Weisel, Zarski, Berger, Krieger, Schaub, Moser, et al.: Efficacy and cost-effectiveness of guided and unguided internet-based mobile-supported indicated prevention of depression and anxiety (ICare Prevent): A multi-country three-armed randomized controlled trial., unpublished). Within the large ICare project, several Internet-based interventions will be used for different mental health conditions. The overall aim is to increase access and uptake of such interventions by overcoming current barriers to implementation and establishing a comprehensive model of mental health promotion in Europe. With the goal of improving quality of healthcare, effectiveness, and acceptance of this kind of intervention, the ICare project addresses core goals of EU health policy.

## Methods/Design

The study is a three-arm, parallel, randomized controlled superiority trial, with an economic evaluation alongside, for college students with subclinical symptoms of depression and/or anxiety. An unguided and a guided version of ICare Prevent will be compared to CAU in a Dutch university setting. Next to screening for eligibility, self-report measures and clinical interviews will be administered at baseline, mid-intervention, and post intervention, as well as at six-month and 12-month follow-ups. The ethics committee of the VU medical center has approved the study (number NL60705.029.17). A populated SPIRIT checklist and figure have been submitted as additional files to this publication (see Additional file 1 for SPIRIT checklist and SPIRIT figure (Additional file 2).

### Participants

Students will be recruited and provided with contact details through e.g. a website (https://icare-online.eu/nl/), social media (e.g. Facebook), and information material distributed on campus at Dutch universities. They are eligible if they: (1) are aged ≥ 16 years; (2) experience at least mild self-reported symptoms of depression, defined as a score ≥ 16 on the Center for Epidemiological Studies Depression scale (CES-D; [41]), and/or anxiety, defined as a score ≥ 5 on the seven-item version of the Generalized Anxiety Disorder scale (GAD-7; [42]); (3) do not meet diagnostic criteria for a mood or anxiety disorder based on the Mini International Neuropsychiatric Interview (M.I.N.I.; [43]) at screening. Exclusion criteria are: (1) being on a waitlist for, currently receiving, or having received psychotherapy over the past six months for any mental health condition; (2) meeting diagnostic criteria for a lifetime bipolar disorder (M.I.N.I.) or having received a diagnosis of a psychiatric disorder; (3) being at moderate to severe risk for suicide (M.I.N.I.); (4) being in remission of a major depressive disorder (MDD) episode, defined as an MDD diagnosis in the previous six months and experiencing at least one cardinal symptom (e.g. persistent and pervasive low mood, loss of interest or pleasure in usual activities) during the previous three weeks (M.I.N.I.); (5) self-reported inability to read or write Dutch; (6) no informed consent; (7) no access to a computer or the Internet; or (8) participating in similar studies at time of inclusion.

### Study procedures

Upon contact, potential participants will receive an information letter and informed consent form by email, the latter to be signed and returned. In addition, the email contains a link to the ICare Prevent platform on which participants have to register. After the informed consent form has been returned, the screening questionnaires for all self-reported inclusion and exclusion criteria will be made available on the platform. The screening consists of: demographic questions; the CES-D; and the GAD-7. Next, eligible participants will be asked for their telephone number in order to conduct the M.I.N.I. interview, the clinician rated version of the Quick Inventory for Depression Scale (QIDS-CR; [44]) and the Structured Interview Guide for the Hamilton Anxiety Rating Scale (SIGH-A; [45]). The interviews will be performed by trained clinical psychology master students under the supervision of a member of the research team; 10% will be recorded and rated by a second rater to assess inter-rater reliability. Consent for the recordings will be asked for at the beginning of the call. Disagreement between raters will be solved by discussion or by asking an experienced psychotherapist. Eligible participants will receive a confirmation by email and access to the baseline self-report questionnaires on the online platform. Once completed, they will be randomized to one of the two intervention conditions or the control group.

Non-eligible participants will be advised to consult their general practitioner or a (student) psychologist if they think they need help for their complaints. Those with moderate to severe suicide risk will also be advised to contact their general practitioner. In addition, they will be informed about the national suicide helpline (113online; [46]). The research team will contact those participants again one week later and ask whether they sought help.

### Randomization, treatment allocation, and blinding

**Fig. 1:**
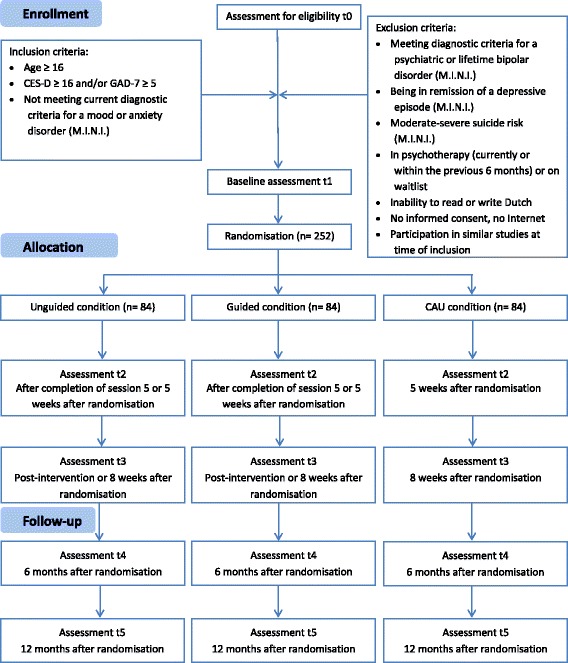
Study flowchart

The allocation scheme will be derived by computer using a random number generator at a 1:1:1 ratio. Participants will be randomized at an individual level by an independent institute (University of Münster, Germany) and randomization will be stratified by type of subclinical symptoms (depression or anxiety). Due to the nature of the study it is not possible to blind either participants or coaches, as they will be notified to which of the three conditions they have been assigned to. However, raters who perform the clinical interviews to assess outcomes will be blinded. In order to ensure that blinding was successful, participants will be informed about the importance not to indicate their allocation status at the beginning of the telephone interviews. Moreover, raters will be asked to guess the allocation status of each participant after the second interview at post intervention. The results will then be compared to what would have been expected by chance. The flowchart of the trial is shown in Fig. 1.

### Online intervention platform

The technology platform used to deliver the ICare Prevent intervention is provided by Minddistrict. This company is full partner of the ICare project consortium and responsible for the provision and maintenance of the platform. Its content management system is used to upload the interventions, add new participants and eCoaches, as well as questionnaires. Access to the platform is provided by means of email and personalized password combination. It is currently used both in clinical research and in routine practice to provide guided and unguided self-help interventions for a variety of mental health conditions. The platform conforms to all required quality standards and operates according to the ISO 27000 and NEN 7510 norms. All data are securely stored on ISO 27000 certified servers and transmitted using HTTPS with SSL certificates (AES-256 and SHA-1, 2048-bit RSA). Unauthorized access to the platform is therefore not possible. A data management and safety plan has been developed as part of the larger ICare project.

### Intervention

ICare Prevent is an Internet-based intervention with mobile-support by means of an application (app). It uses both transdiagnostic and individually tailored components that are based on previously developed Internet-based modules, all using evidence-based CBT principles [47–51]. ICare Prevent consists of seven main sessions and one booster session. Participants are advised to complete one main session per week. The intervention contains text, exercises, images, explanatory videos, as well as audio files and downloadable information. Testimonials are used to illustrate (homework) exercises. The main sessions follow the same basic structure. First, the content of the current session is explained, followed by a review of the homework exercises from the previous session. In accordance with the individually tailored approach of the intervention, participants can choose content depending on their predominant complaints, i.e. depression or anxiety. For example, in the fifth session, participants can either practice problem-solving or exposure techniques. The content per session is summarized in Table 1. Throughout sessions 2–7, participants can choose to complete one of eight choice modules following the main intervention content. In accordance with the transdiagnostic approach of the intervention, these modules aim at increasing resilience and tackle problems common to both depression and anxiety. In addition, five diaries with different content, such as positive activities and sleep, are accessible through the online platform and the app. An overview of all choice modules and diaries is given in Table 2.

**Table 1:** Overview of topics covered by the intervention per session

Session	Topic
1	Introduction, technical aspects, goal-setting, and behavioral activation in the context of basic psychological needs and important personal values
2	Identifying problems and tackling them through behavioral activation
3	Psychoeducation on depression and anxiety
4	Cognitive restructuring and challenging negative thoughts
5	Identifying the most prominent complaints and accordingly follow either:
	a) problem-solving strategies for more prominent depressive complaintsb) exposure strategies for more prominent anxiety complaints
6	Deepening the training on the route chosen in session 5
7	Making a plan for the future
8	Booster session (four weeks after session 7)

**Table 2:** Overview of topics covered by the choice modules and diaries

Choice module	Topic
1	Sleep
2	Perfectionism
3	Gratitude
4	Self-esteem
5	Alcohol consumption (includes AUDIT-C)
6	Relaxation
7	Acceptance
8	Rumination
Diary	Topic
1	Positive activities
2	Negative thoughts
3	Sleep
4	Challenging situations
5	Alcohol consumption

The intervention can be used on (laptop) computers, mobile phones, and tablets. The mobile app can be used optionally. It provides access to the diaries and allows participants to activate push notifications. These short messages contain small exercises, such as short relaxation techniques, as well as motivational texts. The intervention uses a responsive web design in order to tailor content to participants’ needs, based on the choices they make. An optional read-aloud function is included. The expected time to complete each session is approximately 60 min. The two intervention conditions only differ in the support participants receive. In the guided condition, trained eCoaches (clinical psychology master students) spend approximately 20 min, but no more than 30 min per session on sending individual manualized feedback on the exercises through the intervention platform. In addition, they will send reminders for module completion in order to increase adherence. The eCoaches will be supervised by the members of the research team. Participants in the unguided condition receive only automatic motivational messages that aim to increase adherence by positively reinforcing participants for completing intervention modules and reminding them in case of a delay in completion.

### Care as usual (CAU)

Participants in all three conditions may use CAU services. We monitor healthcare services used, as well as other means to alleviate symptoms (e.g. as talking with relatives and friends), by means of a self-report questionnaire, the Client Service Receipt Inventory (CSRI; [52]). In the Netherlands, CAU for subclinical symptoms of depression and/or anxiety can include visits to the general practitioner, making use of support staff trained in the provision of help for mild psychological symptoms, or (student) psychologists according to Dutch guidelines [53, 54]. Alternatives include homeopaths, alternative medicine providers, or self-medication. Participants in the control group will receive access to ICare Prevent one year after trial inclusion.

### Measures

Following screening and baseline (all groups), assessment points for the intervention groups include five weeks after randomization or directly after session 5 (whichever happens first) and eight weeks after randomization or post intervention (whichever happens first). Assessment points for the control group include five and eight weeks after randomization. Follow-up measures for all three groups consist of six and 12 months after randomization. Assessments include self-report questionnaires on the intervention platform and diagnostic interviews administered over the telephone. An overview of all measures taken by assessment point is given in Table 3.

**Table 3:** Overview of instruments with associated assessment points

Measure (instrument)	Assessment point					
	T0	T1	T2	T3	T4	T5
Screening						
Depression (CES-D)	X					
Anxiety (GAD-7)	X		X	X	X	X
Demographic data	X					
Mental disorder diagnosis self-reported	X					
Experience with psychotherapy	X					
Clinical diagnosis (M.I.N.I.)	X				X	X
Primary outcomes						
Depression (QIDS)	X			X	X	X
Anxiety (SIGH-A)	X			X	X	X
Secondary outcomes						
Depression (PHQ-9)		X	X	X	X	X
Anxiety (GAD-7)	X		X	X	X	X
Clinical diagnosis (M.I.N.I.)	X				X	X
Academic performance (PSS, ECTS)		X	X	X	X	X
Cost-effectiveness						
Costs (CSRI)		X			X	X
Other outcomes						
Alcohol use (AUDIT-C)		X	X	X	X	X
Alliance (WAI-SR)^a^			X			
Behavioral activation (BADS-SF)		X		X	X	X
Reasons for dropout^a^				X		
Expectations (CEQ)		X				
Incongruence (INKK)		X		X	X	X
Motivation (TEQ)		X	X	X		
Negative effects of treatment (INEP)^a^				X		
Personality (BFI-10)		X				
Potential risk factors^b^		X				
Program evaluation (CSQ-8)^a^			X	X		
Quality of life (AQoL, EQ-5D-8 L)		X		X	X	X
Resilience (CD-RISC)		X	X	X	X	X
Sleep quality (PSQI)		X		X	X	X
Support^a^			X			
Wellbeing (WHO-5)		X		X	X	X
Worry (PSWQ-3)		X		X	X	X

^a^Only in intervention groups

^b^Optional

*T0* screening, *T1* baseline, *T2* after completion of session 5 or 5 weeks after randomization, *T3* post intervention, *T4* 6-month follow-up, *T5* 12-month follow-up

### Screening

#### Depression

The CES-D [41] will be used as a screening instrument. It has been shown to be reliable and valid in studies across different populations, including students [55, 56].

#### Anxiety

The GAD-7 [42] will be used both as screening as well as outcome measure. It has been tested in students as well as different age groups and showed excellent reliability and validity [57, 58].

#### Demographic data, self-reported mental disorder diagnosis, and experience with psychotherapy

In addition to demographic data, such as age, participants indicate if they have ever received a diagnosis of any, and, if so, which, mental health disorder and whether they have ever utilized psychological treatment for any mental health condition.

#### Diagnostic interview

The M.I.N.I. [43] will be administered by telephone to establish DSM-V diagnoses of mood and anxiety disorders, bipolar disorder, psychosis, and the risk for suicide during screening. The reliability and validity of the M.I.N.I. has been established among several populations [59, 60].

### Primary outcome

The primary outcome is change in disorder-specific symptom severity from baseline to post intervention. For depression, this will be assessed using the QIDS-CR [44]. This interview has been used in different settings and has good psychometric properties [44, 61]. The SIGH-A [45] will be used for the assessment of anxiety. Its reliability and validity has been found to be good [45].

### Secondary outcomes

Secondary outcomes include self-reported reduction in depressive and anxiety symptoms from baseline to post intervention. In addition to screening, the GAD-7 will be used to measure anxiety symptoms throughout the trial. The nine-item Patient Health Questionnaire (PHQ-9; [62]) will be used to assess depressive symptoms. Research on the PHQ-9 has generally shown good psychometric properties among all age groups [62–64]. Moreover, the time to onset of a mood or anxiety disorder within the 12-month follow-up period will be assessed by the M.I.N.I. Finally, academic performance will be measured by the Presenteeism Scale for Students (PSS; [65]). It is a valid and reliable measure that will be used to assess presenteeism in the student population [65]. In addition, students will be asked to provide the number of points they acquired on the European Credit Transfer System (ECTS) during the semester.

### Costs

The CSRI [52] will be used to track societal costs. It has been adapted to the specifics of the Dutch college student context, measuring costs associated with healthcare use. In addition, it assesses absenteeism and presenteeism in college as well as in student jobs. For the latter, the Dutch minimum wage will be taken as an estimate of students’ income.

### Other outcomes, moderators, and mediators

#### Alcohol use

Alcohol use will be monitored by the three consumption questions of the brief Alcohol Use Disorders Identification Test (AUDIT-C; [66]). The AUDIT-C has shown good psychometric properties in college students [67]. Moreover, it has been used both as a screening as well as outcome instrument [68, 69].

#### Alliance (only intervention arms)

An adapted version of the self-rated Working Alliance Inventory (WAI-SR; [70]) will be administered to the active intervention groups. Questions relating to the bond with the coach will be asked only in the guided condition. The original form of the WAI-SR has shown good psychometric properties [70, 71].

#### Behavioral activation

The short form of the Behavioral Activation for Depression Scale (BADS-SF; [72, 73]) assesses activation and engagement in pleasant activities. The reliability and validity of the questionnaire has been established in a college student sample [74].

#### Dropout

Participants will be asked whether and why they have prematurely stopped using the intervention.

#### Expectations (only intervention arms)

The Credibility/Expectancy Questionnaire (CEQ; [75]) will be used to assess participants’ expectations of the intervention. Its reliability and validity has been established and the questionnaire has been used in college student samples [75, 76].

#### Incongruence

ICare Prevent aims at reducing the gap between motivational goals and their actual execution. The short version of the incongruence questionnaire (INKK; [77]) measures the extent to which this is achieved.

#### Motivation

The Treatment Entry Questionnaire (TEQ; [78]) will be used to assess motivation to participate in the study. Research has indicated good psychometric properties for the TEQ in a Dutch psychiatric sample [79].

#### Negative effects of the intervention (only in intervention arms)

The Inventory for the Assessment of Negative Effects of Psychotherapy (INEP; [80]) measures possible negative aspects of using the intervention.

#### Personality

The ten-item Big Five Inventory (BFI-10; [81, 82]) will be used as a brief measure of personality. Its psychometric properties have been established in a college student sample [82].

#### Potential risk factors

Participants can choose if they want to answer a battery of potential risk factors for mental wellbeing that have been suggested by the literature, such as smoking, body image, and childhood abuse.

#### Program evaluation (only in intervention arms)

The eight-item Client Satisfaction Questionnaire has been adapted to this online context (CSQ-8; [83, 84]). It assesses participants’ satisfaction with the intervention. The psychometric properties of the Dutch version have been found to be good [85].

#### Quality of life

In addition to the Assessment of Quality of Life questionnaire (AQoL-8D; [86, 87]), the EuroQol (EQ-5D-5 L; [88]) will be administered. This enables the calculation of quality-adjusted life-years (QALYs) and converting health states into utility scores using the Dutch EQ-5D-5 L tariff [89]. Both instruments have established psychometric properties [86, 90].

#### Resilience

The ten-item version of the Connor-Davidson Resilience Scale (CD-RISC; [91]) measures how good individuals can work under pressure or how strong they think they are. The CD-RISC has been used in college samples and its reliability and validity has been well established in different populations [92–94].

#### Sleep quality

One item on sleep quality from the Pittsburgh Sleep Quality Index (PSQI; [95]) will be administered. The PSQI has been used extensively in college samples [96, 97].

#### Support (only in guided intervention arm)

Seven items have been developed by the research team to assess the relationship between participants and their eCoach (e.g. “I think my eCoach appreciates me less if I use ICare Prevent less often than expected”), as well as how they would rate the eCoach’s competency (e.g. “I think my eCoach is very competent”).

#### Wellbeing

Wellbeing will be measured by the five-item version of the World Health Organization Ten Well-Being Index (WHO-5; [98]). Good psychometric properties of this instrument have been established in a college and Dutch population [99, 100].

#### Worry

The ultra-brief version of the Penn State Worry Questionnaire (PSWQ-3; [101]) has good psychometric properties and will be administered to examine excessive worrying [101, 102].

### Sample size calculation

The primary endpoint is change in disorder specific symptom severity from baseline to post intervention. Based on the evidence from a meta-analysis by Cuijpers et al. [103] on the effectiveness of psychotherapies for subclinical symptoms of depression, as well as a randomized controlled trial on the effectiveness of an Internet-based intervention for subclinical depression ([24–26], the expected effect size is *d* = 0.35. Due to a lack of information on effect sizes for subclinical symptoms of anxiety, *d* = 0.25 is used as a conservative estimation for both conditions. A study comparing the effectiveness of Internet-based psychotherapy with different levels of support found effect sizes in a similar range [39]. The sample size calculation is based on a repeated measure ANOVA and follows the recommendations given in Muller [104] and Muller et al. [105]. Thus, based on a global significance level α = 0.05 and power β = 0.95, 252 participants (84 per study arm) will be needed for an effect of 0.25 to be significant.

### Statistical analysis

All analyses for the primary and secondary outcomes will be based on multilevel mixed model regression analyses for continuous outcomes in order to assess change over time. Time will be used as a predictor variable and baseline measures will be included as covariates. Linear models will be used for normally distributed data and negative binomial models for left-skewed data. All analyses will be performed on an intention-to-treat (ITT) sample. Per-protocol analyses including only participants who conformed to their group allocation will be performed in addition. Missing data will be handled using either multiple regression imputation techniques if appropriate or full information maximum likelihood estimation [106]. The secondary outcome, time to mental health disorder onset during 12 months follow-up in all conditions, measured by the M.I.N.I., will also be compared using survival curve analysis. The curves will be compared using cox proportional hazard regression analysis using baseline symptom severity as a covariate. Hazard ratios will be calculated as measure of the effect size of group differences. Within secondary analyses, interactions with predictors will be added to the model in order to identify risk and protective factors.

### Cost-effectiveness

Cost-effectiveness will be assessed from a societal perspective for both disorder-free days and QALYs. The analysis will be performed according to the ITT principle. Multiple imputation according to the MICE algorithm developed by Van Buuren et al. [107] will be used to impute missing cost and effect data. Incremental cost-effectiveness ratios (ICERs) will be calculated by dividing the pairwise differences in the mean societal costs between the groups by the pairwise differences in mean effects between the groups. Bivariate regression models will be used to estimate cost and effect differences while adjusting for confounding if necessary. Statistical uncertainty will be estimated using bias-corrected accelerated bootstrapping with 5000 replications. Uncertainty surrounding the ICERs will be presented in cost-effectiveness planes and acceptability curves [108].

## Discussion

The high prevalence and burden of depression and anxiety, both on an individual and societal level, warrant effective and low-threshold prevention strategies. Students are considered an at-risk group for developing these conditions due to their age of onset and the challenges that this population faces [6]. So far, research on the effectiveness of Internet-based prevention in college students is largely limited to the field of eating disorders and substance use [109, 110]. However, scientific evidence on the value of, in particular, transdiagnostic individually tailored interventions for the indicated prevention of depression and anxiety among college students is scarce.

Based on these considerations, we have developed the ICare Prevent intervention. Originally conceived for the indicated prevention in the general population in Germany, we have adapted it to a Dutch college student context. To do that, we have started with a literal translation from German into Dutch. In addition, focus groups have been conducted with students to map out core features that are attractive to them. As a result, the intervention has been shortened considerably while leaving the psychotherapeutic components intact. This was assured by the supervision of a licensed psychotherapist. Moreover, problem descriptions and the testimonials have been changed to represent the diverse student population and their experiences related to study stress, balancing work and studies, as well as dealing with family members, partners, and friends.

### Strengths and limitations

To our knowledge, this is the first trial that investigates the (cost-)effectiveness of a transdiagnostic individually tailored Internet-based intervention for the prevention of depression and anxiety in a college student population. Data on both symptom reduction as well as the time to onset of a mood or anxiety disorder will be measured. Although some research suggests that Internet-based interventions have the potential to be cost-effective compared to no treatment, no data are available for preventive transdiagnostic individually tailored Internet-based interventions for college students. Specifically, the added (economic) value of support by means of an eCoach needs to be closely monitored in the context of this low-intensity intervention.

Some possible limitations of the present trial should be noted. First, the sample size of this trial conducted among Dutch college students is insufficient for directly comparing the guided and unguided treatment arm as well as analyzing the time to a mental health disorder onset. Similarly, analyses of moderators and mediators will be underpowered and may therefore provide only limited insights. To counter this issue, data from all participating trials in the ICare project will be pooled in order to achieve enough statistical power. Second, an evaluation of societal costs might be difficult due to the fact that most students are seldom in full-time employment. The detrimental effect of depression and anxiety on absenteeism and presenteeism in the context of employment is therefore difficult to quantify. In order to increase the validity of the results, we have adapted the cost-effectiveness questionnaire (CSRI; [52]) to fit the specifics of a student population by focusing on absenteeism and presenteeism in student jobs, as well as in lectures. Finally, previous research has shown that adherence to Internet interventions is a point of concern, especially in unguided formats [111, 112]. A series of automatic motivational messages and reminders will be used in order to increase adherence in the unguided study arm and we will closely observe factors related to dropout. This will be beneficial in designing future interventions and improving uptake of intervention content.

## Trial status

Recruitment started in July 2017. Follow-up assessments for the last participant are expected to be completed by 31 August 2019.

## Additional files

Additional file 1: A populated SPIRIT checklist. (PDF 95 kb)

Additional file 2: SPIRIT figure. (PDF 28 kb)

## Abbreviations

AES: Advanced encryption standard
AQoL-8D: Assessment of Quality of Life – 8 Dimension version
AUDIT-C: Alcohol Use Disorders Identification Test – Consumption questions
BADS-SF: Behavioral Activation for Depression Scale – Short Form
BFI-10: Big Five Inventory – 10 item version
CAU: Care as usual
CBT: Cognitive behavioral therapy
CD-RISC: Connor Davidson Resilience Scale
CEQ: Credibility/Expectancy Questionnaire
CES-D: Center for Epidemiologic Studies Depression Scale
CSQ-8: Client Satisfaction Questionnaire – 8 item version
CSRI: Client Service Receipt Inventory
ECTS: European Credit Transfer System
EQ-5D-5 L: EuroQol – 5 Dimension – 5 Level questionnaire
GAD-7: Generalized Anxiety Disorder Scale – 7 item version
HTTPS: Hyper text transfer protocol secure
ICER: Incremental cost-effectiveness ratio
INEP: Inventory of Negative Effects of Psychotherapy [Inventar zur Erfassung negativer Effekte von Psychotherapie]
INKK: Incongruence Questionnaire – Short Form [Inkonkruenzfragebogen - Kurzform]
ISO: International Organization for Standardization
ITT: Intention-to-treat
M.I.N.I.: Mini International Neuropsychiatric Interview
MDD: Major depressive episode
NEN: Dutch norm [NEderlandse Norm]
PHQ-9: Patient Health Questionnaire – 9 item version
PSQI: Pittsburgh Sleep Quality Index
PSS: Presenteeism Scale for Students
PSWQ-3: Penn State Worry Questionnaire – 3 item version
QALY: Quality-adjusted life years
QIDS-CR: Quick Inventory for Depression Scale – Clinician Rated
RSA: Asymmetric Encryption Algorithm
SHA-1: Secure Hash Algorithm 1
SIGH-A: Structured Interview Guide for the Hamilton Anxiety Rating Scale
SSL: Secure sockets layer
TEQ: Treatment Expectancy Questionnaire
WAI-SR: Working Alliance Inventory – Self-rated
WHO-5: World Health Organization Ten Well-being Index – 5 item version
