# Supplementary material for: Effectiveness of a transdiagnostic individually tailored Internet-based and mobile-supported intervention for the indicated prevention of depression and anxiety (ICare Prevent) in Dutch college students: study protocol for a randomised controlled trial
Source: Trials. 2018 Feb 20;19:118. doi: 10.1186/s13063-018-2477-y (PMC5819200; doi:10.1186/s13063-018-2477-y)
Supplement: Supplementary file 2 — SPIRIT figure. (PDF 28 kb) [file 13063_2018_2477_MOESM2_ESM.pdf]

SPIRIT Figure**.** Schedule of screening/enrolment, interventions, and assessments.

<table>
<tr><th></th><th colspan="7">STUDY PERIOD</th></tr>
<tr><th></th><th colspan="2">Screening/ enrollment</th><th colspan="3">Post-allocation</th><th colspan="2">Follow-up</th></tr>
<tr><th>TIMEPOINT</th><th>$-t_1$</th><th>$t_0$</th><th>$t_1$</th><th>$t_2$</th><th>$t_3$</th><th>$t_4$</th><th>$t_5$</th></tr>
<tr><td>SCREENING AND ENROLMENT:</td><td></td><td></td><td></td><td></td><td></td><td></td><td></td></tr>
<tr><td>Informed consent (before eligibility screen)</td><td>X</td><td></td><td></td><td></td><td></td><td></td><td></td></tr>
<tr><td>Eligibility screen</td><td>X</td><td></td><td></td><td></td><td></td><td></td><td></td></tr>
<tr><td>Enrollment</td><td></td><td>X</td><td></td><td></td><td></td><td></td><td></td></tr>
<tr><td>INTERVENTIONS:</td><td colspan="2"></td><td></td><td></td><td></td><td></td><td></td></tr>
<tr><td>Guided ICare Prevent</td><td colspan="2"></td><td colspan="3">◆————◆</td><td></td><td></td></tr>
<tr><td>Unguided ICare Prevent</td><td colspan="2"></td><td colspan="3">◆————◆</td><td></td><td></td></tr>
<tr><td>CAU</td><td colspan="2"></td><td>X</td><td>X</td><td>X</td><td></td><td></td></tr>
<tr><td>ASSESSMENTS:</td><td colspan="2"></td><td></td><td></td><td></td><td></td><td></td></tr>
<tr><td>Depression (CES-D)</td><td colspan="2">X</td><td></td><td></td><td></td><td></td><td></td></tr>
<tr><td>Depression (QIDS)</td><td colspan="2">X</td><td></td><td></td><td>X</td><td>X</td><td>X</td></tr>
<tr><td>Depression (PHQ-9)</td><td colspan="2"></td><td>X</td><td>X</td><td>X</td><td>X</td><td>X</td></tr>
<tr><td>Anxiety (GAD-7)</td><td colspan="2">X</td><td></td><td>X</td><td>X</td><td>X</td><td>X</td></tr>
<tr><td>Anxiety (SIGH-A)</td><td colspan="2">X</td><td></td><td></td><td>X</td><td>X</td><td>X</td></tr>
<tr><td>Demographic data</td><td colspan="2">X</td><td></td><td></td><td></td><td></td><td></td></tr>
<tr><td>Mental disorder diagnosis self-reported</td><td colspan="2">X</td><td></td><td></td><td></td><td></td><td></td></tr>
<tr><td>Experience with psychotherapy</td><td colspan="2">X</td><td></td><td></td><td></td><td></td><td></td></tr>
<tr><td>Clinical diagnosis (M.I.N.I.)</td><td colspan="2">X</td><td></td><td></td><td></td><td>X</td><td>X</td></tr>
<tr><td>Academic performance (PSS, ECTS)</td><td colspan="2"></td><td>X</td><td>X</td><td>X</td><td>X</td><td>X</td></tr>
<tr><td>Costs (CSRI)</td><td colspan="2"></td><td>X</td><td></td><td></td><td>X</td><td>X</td></tr>
</table>

| | | | | | | |
|---|---|---|---|---|---|---|
| ***Alcohol use (AUDIT-C)*** | | X | X | X | X | X |
| ***Alliance (WAI-SR) **** | | | X | | | |
| ***Behavioral activation (BADS-SF)*** | | X | | X | X | X |
| ***Reasons for dropout **** | | | | X | | |
| ***Expectations (CEQ)*** | | X | | | | |
| ***Incongruence (INKK)*** | | X | | X | X | X |
| ***Motivation (TEQ)*** | | X | X | X | | |
| ***Negative effects of treatment (INEP) **** | | | | X | | |
| ***Personality (BFI-10)*** | | X | | | | |
| ***Potential risk factors **** | | X | | | | |
| ***Program evaluation (CSQ-8) **** | | | X | X | | |
| ***Quality of life (AQoL, EQ-5D-8L)*** | | X | | X | X | X |
| ***Resilience (CD-RISC)*** | | X | X | X | X | X |
| ***Sleep quality (PSQI)*** | | X | | X | X | X |
| ***Support **** | | | X | | | |
| ***Wellbeing (WHO-5)*** | | X | | X | X | X |
| ***Worry (PSWQ-3)*** | | X | | X | X | X |

*Note.* $t_{-1}$= Informed consent and subsequent screening; $t_0$= Enrollment; $t_1$= Baseline; $t_2$= after completion of session 5 or 5 weeks after randomisation; $t_3$= Post-intervention, $t_4$= 6 month follow-up; $t_5$= 12 month follow-up

*= optional; ** = only in intervention groups
